# Simultaneous co-localized super-resolution fluorescence microscopy and atomic force microscopy: combined SIM and AFM platform for the life sciences

**DOI:** 10.1038/s41598-020-57885-z

**Published:** 2020-01-24

**Authors:** Ana I. Gómez-Varela, Dimitar R. Stamov, Adelaide Miranda, Rosana Alves, Cláudia Barata-Antunes, Daphné Dambournet, David G. Drubin, Sandra Paiva, Pieter A. A. De Beule

**Affiliations:** 1 International Iberian Nanotechnology Laboratory, Avenida Mestre José Veiga s/n, Braga, Portugal; 2 Department of Applied Physics, University of Santiago de Compostela, E-15782 Santiago de Compostela, Spain; 3 JPK BioAFM Business, Nano Surfaces Division, Bruker Nano GmbH, Am Studio 2D, 12489 Berlin, Germany; 4 Centre of Molecular and Environmental Biology, Department of Biology, University of Minho, Braga, Portugal; 5 Department of Molecular and Cell Biology, University of California, Berkeley, Berkeley, CA USA

**Keywords:** Biological physics, Atomic force microscopy, Super-resolution microscopy

## Abstract

Correlating data from different microscopy techniques holds the potential to discover new facets of signaling events in cellular biology. Here we report for the first time a hardware set-up capable of achieving simultaneous co-localized imaging of spatially correlated far-field super-resolution fluorescence microscopy and atomic force microscopy, a feat only obtained until now by fluorescence microscopy set-ups with spatial resolution restricted by the Abbe diffraction limit. We detail system integration and demonstrate system performance using sub-resolution fluorescent beads and applied to a test sample consisting of human bone osteosarcoma epithelial cells, with plasma membrane transporter 1 (MCT1) tagged with an enhanced green fluorescent protein (EGFP) at the N-terminal.

## Introduction

Visualization of biological specimens can be accomplished through several available microscopy techniques, each of them presenting inherent benefits and constraints. The combination of different imaging platforms can further extend their applications^1,2^. The major significance of correlative imaging relies on its capacity to provide complementary information such as chemical and biophysical details from the same sample during a single experiment. Atomic Force Microscope (AFM) was introduced in 1986 by Binning *et al*.^3^, being a high-resolution surface probe technique that allows topographical and nanomechanical characterization. AFM has become a powerful tool to team up with optical microscopes, especially regarding the biosciences field. On the other hand, super-resolution fluorescence optical microscopy methods have been a major breakthrough for biological samples examination, having been actively developed in the latter years to surpass the optical diffraction barrier^4^. Nonetheless, they are limited in some aspects such as for example nanomechanical sample characterization. Therefore, a correlative microscopy approach is beneficial as it allows to pair up the high spatial resolution and mechanical characterization potential of an AFM, with the additional molecular information available, through various fluorescent markers or immunostains^5,6^.

Despite the great potential on simultaneous co-localized AFM imaging and super-resolution fluorescence microscopy, no one to the best of our knowledge has reported it. Current AFM techniques combining optical fluorescence imaging normally perform successive measurements and then superimpose the images obtained from the separate acquisitions. Main reasons for this are: (i) imaging in a simultaneous mode causes perturbation on the AFM cantilever operation due to fluorescence excitation light, and (ii) noise transfer from the optical microscope can disturb AFM measurements^7,8^.

The atomic force microscope has been successfully integrated with different optical microscopy techniques^9–11^, allowing to overcome some of its individual limitations when it comes to, for instance, sample penetration and biological specificity. In this regard, AFM has been combined with Confocal Laser Scanning Microscopy (CLSM)^12–14^, Aperture Correlation Microscopy^8^, Total Internal Reflection Fluorescence Microscopy (TIRFM)^15–21^, and Fluorescence Lifetime Imaging (FLIM)^22,23^. AFM has also been integrated with far-field super-resolution microscopy schemes, namely Stimulated Emission Depletion (STED)^24–27^, Photoactivated Localization Microscopy (PALM)^28^ and Stochastic Optical Reconstruction Microscopy (STORM)^29,30^. The integration of AFM with fluorescence super-resolution schemes faces a variety of problems impeding simultaneous imaging data acquisition. For instance, high-powered light sources interact strongly with gold coated AFM cantilevers, causing excessive heating or even cantilever coating deterioration. Such high-powered light sources are commonplace for the depletion beam of a STED set-up. Nonetheless, Chacko et al. demonstrated simultaneous STED imaging and nanomanipulation by AFM^31^.

In STORM microscopy high-powered lasers are also present as accelerators for a fast read-out. Furthermore, STORM microscopy requires fluorophores exhibiting blinking behaviour which is typically promoted by immersing the sample in a buffer containing an enzymatic oxygen scavenger. The latter precludes simultaneous operation of the AFM and fluorescent measurements, as the ingredients of the buffer stick to the AFM cantilever, so correlative imaging is normally performed by acquiring first the AFM image and then adding the buffer for super-resolution microscopy^28,32^. Recent research has proposed a new dye to circumvent this problem that allows correlative AFM and STORM imaging without the need to change the buffer^30^.

The efforts to increase the AFM imaging rate have resulted on the development of High-Speed AFM (HS-AFM). HS-AFM has allowed the simultaneous assessment of structure and dynamics of single proteins during their functional activity^33,34^. In order to extend the applications of HS-AFM, it has been combined with light microscopy techniques. Simultaneous imaging capabilities of HS-AFM and Total Internal Reflection Microscopy (TIRFM) were demonstrated in 2013^35^. Scanning Near-Field Optical Microscopy (SNOM) is also a candidate for integration with HS-AFM. Recently, simultaneous tip-enhanced TIRFM/HS-AFM and HS-SNOM/HS-AFM imaging at high spatiotemporal resolution studies have been reported^36^. These microscopy systems open a new avenue on the versatility of HS-AFM for life science research applications.

Here, we report on a new microscopy platform that allows to perform simultaneously super-resolution fluorescence microscopy and atomic force microscopy. In this context, we will refer to simultaneous operation of both techniques as the capability to acquire AFM data under constant SIM illumination without inducing distortions on the AFM cantilever. Super-resolution fluorescence imaging and AFM imaging were performed concomitantly. We combine AFM with Super-Resolved Structured Illumination Microscopy (SR-SIM), a super-resolution technique that allows to surpass the diffraction barrier by a factor of two in every spatial direction^37,38^. Contrary to other super-resolution modalities, SR-SIM relies on a fluorescent excitation light pattern favourable to avoid AFM cantilever disruption during simultaneous operation. The principle of SR-SIM relies on the projection of a grating onto the sample and the subsequent reconstruction of the high-resolution image from different grating imaging positions. This technique has been used for biological imaging, in particular for fixed samples, but it has also proven to be easily applicable to live cell imaging^39,40^. SR-SIM is a very promising imaging modality to reveal dynamic processes in live biological samples in 3D and presents some advantages over other super-resolution techniques as it does not require the use of specialized fluorophores or sample preparations, induces less phototoxicity and can reach higher acquisition rates for longer periods of time as compared to PALM/STORM and STED^41^. SR-SIM images are obtained usually in a two-step process where the raw data have to be subsequently reconstructed. This provokes that the SR-SIM images are not produced with the same immediacy as other techniques such as confocal microscopy. A recent advance in this regard has been done by Markwirth *et al*., who have demonstrated a multicolor structured illumination microscope capable of video-rate imaging^42^. Because SR-SIM requires a relatively low illumination power, its integration with AFM is very appropriate for non-disruptive simultaneous operation. Aside, as SR-SIM is subject to suffer from artefacts during image reconstruction process^43,44^, a combination with AFM can help to validate super-resolution data.

To demonstrate system performance, we first image a sample consisting of sub-resolution fluorescent beads. SR-SIM/AFM simultaneous operation is then explored using CRISPR/Cas9 genome-edited human cells expressing a plasma membrane transporter, MCT1, tagged with EGFP fluorescent protein. This model of EGFP-MCT1 expressing cells was used as a biological test sample and a proof of principle, being particularly relevant for super-resolution microscopy techniques since it avoids the presence of overexpression-induced artefacts that often affect protein localization and dynamic investigations.

## Optical Set-up For Combined SR-SIM and AFM

**Figure 1:**
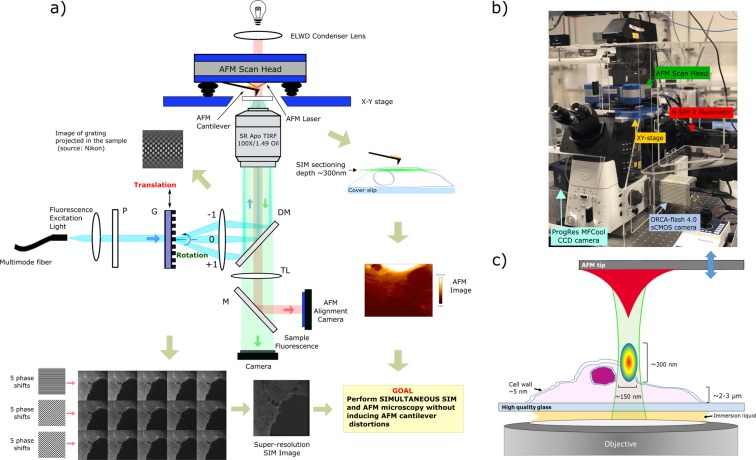
SR-SIM/AFM system. (**a**) Simplified schematic set-up of the SR-SIM/AFM system. In this case, structured illumination is generated using a grating (G). Collimated and expanded laser light illuminates the grating, resulting in the diffraction of multiple orders. Only 0^th^ and ± 1^st^ orders are allowed into the illumination path and focused on the back focal plane of the objective and the created stripped illumination pattern excites the sample. Collection of the fluorescence signal is achieved by a high aperture microscope objective, a dichromatic mirror (DM) and a tube lens (TL). The AFM laser is reflected by a mirror (M) to a high-dynamic range camera coupled to a microscope port for cantilever laser spot alignment. At the same time, nanomechanical mapping of the sample is performed by a probe consisting of a flexible cantilever and a tip. After simultaneous SIM and AFM images acquisition, they are combined into a single image. (**b**) Image of the SR-SIM/AFM system. The N-SIM illuminator module provides the structured illumination. AFM scan head (XYZ) and sample positioning stage (XY) are mounted on the inverted optical microscope. Laser spot alignment of the AFM is accomplished using a standard monochrome CCD camera while fluorescence signal is acquired with a scientific CMOS camera. (**c**) Schematic illustration of the simultaneous SR-SIM/AFM imaging conditions within the sample region. SR-SIM allows to observe the fluorescence signal with a two-fold enhancement in lateral and axial resolutions compared to widefield microscopy. 3D-SIM mode generates an illumination pattern on the sample that is very favourable for integration with AFM without inducing cantilever distortions. Under this illumination, AFM quantitative imaging mode measures a force-distance curve at every pixel of the image.

The combined SR-SIM/AFM platform is based on an atomic force microscope (JPK NanoWizard 3, Bruker Nano GmbH, Berlin, Germany) and a structured illumination microscope (N-SIM E, Nikon Instruments Europe B.V., Amsterdam, The Netherlands). The schematic set-up of the system is shown in Fig. 1a. The AFM is mounted on an inverted microscope (Eclipse Ti2-E, Nikon Instruments Europe B.V., Amsterdam, The Netherlands) and a standard monochrome CCD camera (ProgRes MFCool, Jenoptik, Jena, Germany) is coupled for AFM laser spot cantilever alignment. An infrared laser filter is mounted on the binocular phototube of the Ti2 body for maintaining ocular safety and an extra long working distance (ELWD) condenser of 75 mm (T1-CELWD ELWD, Nikon Instruments Europe B.V., Amsterdam, The Netherlands) is installed in the system.

**Figure 2:**
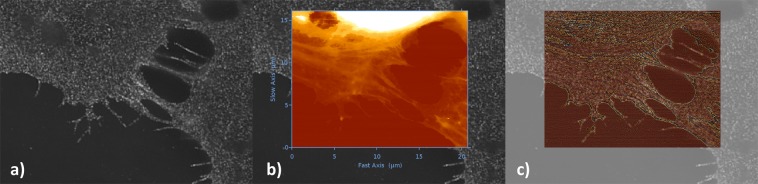
Correlation of the SR-SIM and AFM data. The SR-SIM images of the human bone osteosarcoma epithelial cells (**a**) are superimposed with the height/topography channel of the AFM data (**b**). The correlated SR-SIM/AFM channels on cells in a semi-transparent mode (**c**), include a mean-curvature enhanced AFM-topographic information (see details in text). XY-scales of the AFM image in (**b**) are 20.74 *μ*m and 16.12 *μ*m respectively, recorded at a resolution of 256 × 199 pixels. SR-SIM images were obtained using NIS-Elements (v5.11.00), while the overlay of the SR-SIM and AFM data were generated with the JPK Data Processing software (v6.1.120).

**Figure 3:**
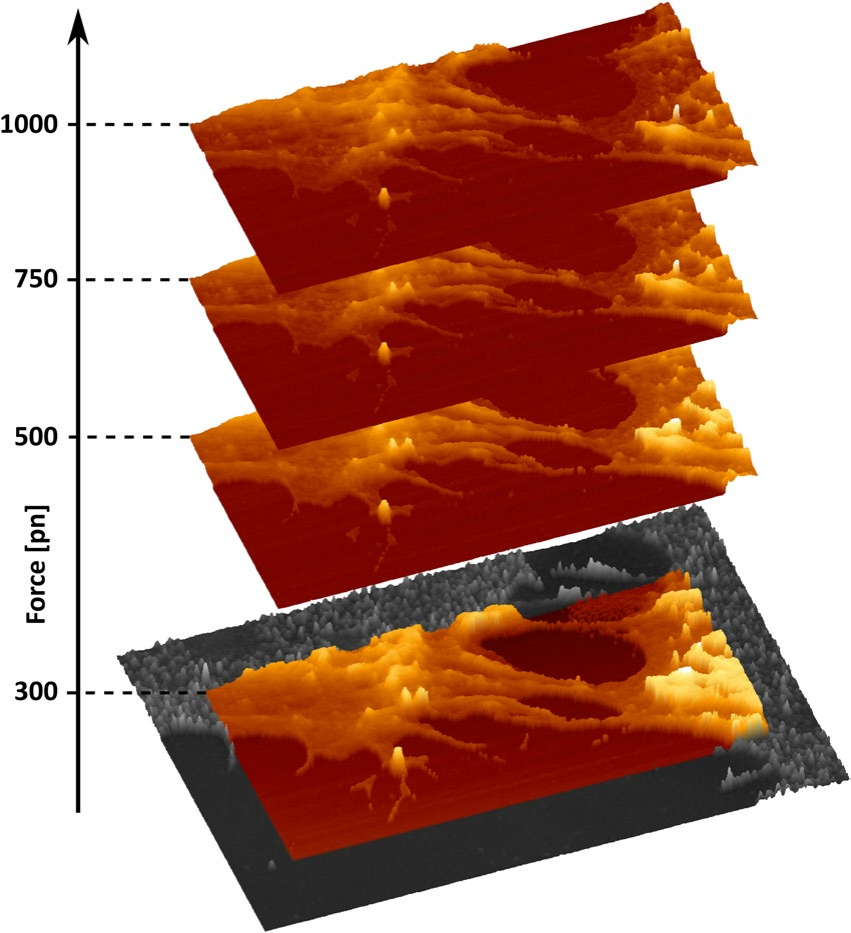
Multidimensional representation of correlated microscopy sets. Recording a complete set of force curves enables the calculation of the sample topography within the range of pre-defined force setpoint and reconstruct the surface structure of the cells. The overlay at 300 pN with the SR-SIM channel is given for consideration of the spatial overlay accuracy. XY-scales of the AFM renders are 20.74 *μ*m and 10.37 *μ*m respectively, recorded at a resolution of 256 × 128 pixels. SR-SIM images were obtained using NIS-Elements (v5.11.00), while the overlay of the SR-SIM and AFM data were generated with the JPK Data Processing software (v6.1.120).

**Figure 4:**
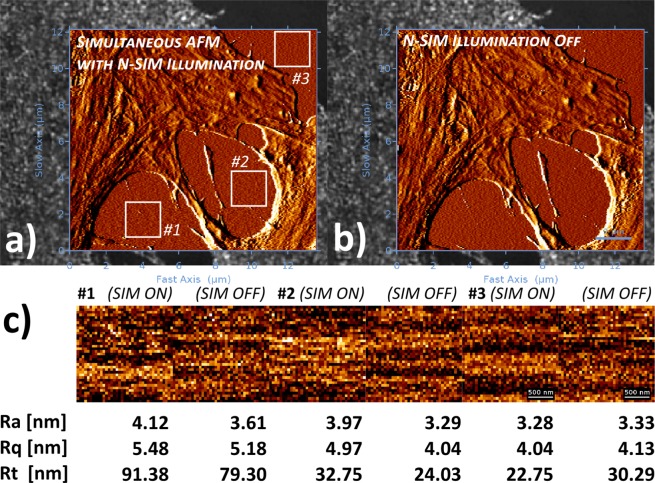
Simultaneous SR-SIM/AFM acquisition. The AFM measurements were carried out on fixed U2OS cells in medium/buffer with (**a**) and without N-SIM illumination (**b**). For convenience and enhanced feature/noise contrast, both AFM topography images in the SR-SIM/AFM overlays are displayed with an edge detection algorithm using a pixel difference operator in X. The topography images from Petri dish surface on three positions (labelled in the figures) were planefit (1^st^ order polynomial function) to compensate for tilts in the sample surface, and subjected to surface roughness analysis (**c**). For comparison reasons, the average roughness (Ra), RMS roughness (Rq) and peak-to-valley roughness (Rt) values are given below the corresponding height profiles. XY-scales of the AFM images in (**a**,**b**) are 13.59 *μ*m and 12.16 *μ*m respectively, recorded at a resolution of 256 × 229 pixels. The insets used for analysis in (**c**) have a resolution of 37 × 37 pixels. SR-SIM images were obtained using NIS-Elements (v5.11.00), while the overlay of the SR-SIM and AFM data were generated with the JPK Data Processing software (v6.1.120).

The AFM was operated in an advanced force-spectroscopy based mode, referred to as Quantitative Imaging (QI), which allows nanomechanical characterization and simultaneous imaging^45^. For static measurements on beads we used force modulation cantilevers (FM, NanoWorld, Switzerland), with a nominal resonance frequency of 75 kHz in air, spring constant of 2.8 Nm^−1^, reflective detector gold coating, and monolithic silicon pyramidal tips with radius of curvature (ROC) of 8 nm. For measurements on cells in liquid, we used qp-BioAC-CI-CB1 cantilevers (NanoSensors, Switzerland), with a nominal resonance frequency of 90 kHz (in air), spring constant of 0.3 Nm^−1^, partial gold coating on the detector side, and quartz-like circular symmetric hyperbolic (double-concaved) tips with ROC of 30 nm. The corresponding AFM areas for the cell images were acquired with a Z-cantilever velocity of 250 μms^−1^ at a max Z-length of 1.5 *μ*m, resulting in an acquisition time (based on the number of pixels) for Figs. 2, 3, 4 of ca. 13, 8 and 15 min respectively.

SIM illumination is provided by laser light previously coupled into a multimodal fiber. In the Nikon N-SIM E microscope three different wavelengths are available (488/561/640 nm). The output collimated light from the fiber travels towards a grating. Only the zero, first positive, and first negative order diffracted light (3D SIM mode) are allowed to pass by a blocking element, resulting in a grating projected onto the sample that fades away into a blur a short distance away from focus, favourable for providing constant illumination of the AFM cantilever. For fluorescence light collection, a high aperture microscope objective (CFI SR APO TIRF 100 × Oil N.A. 1.49, WD 0.12 mm, Nikon Instruments Europe B.V., Amsterdam, The Netherlands) is used. Super-resolution information is recorded with a scientific CMOS (sCMOS) camera (C11440-42U Orca Flash4.0 LT, Hamamatsu Photonics K.K., Boston, MA). The final image is reconstructed by computational methods (NIS-Elements v5.11.00, Nikon Instruments Europe B.V., Amsterdam, The Netherlands). The reconstruction process uses 15 raw images obtained at different orientations of the structured illumination, which is done by moving the diffraction grating (5 phase shifts and 3 rotations around the optical axis). The EGFP-MCT1 expressing cells were imaged with 700 ms exposure time per raw frame. All elements are controlled with NIS Elements AR from Nikon Instruments.

The entire platform (Fig. 1b) is mounted on an optical table with active isolator leg bundles (T1225Q - Nexus Optical Table, Thorlabs, Inc., Newton, NJ, USA). All experiments were carried out at 21 °C. A schematic illustration of the experimental set-up within the sample area is shown in Fig. 1c.

## Results and Discussion

Once it was verified that it is possible to work below the diffraction limit (see SR-SIM resolution enhancement), we proceeded with correlation of the optical and AFM data. The accuracy of the optical overlay relies on the XY-linearized closed loop positioning of the AFM piezos. By moving the tip to a set of fixed coordinates (3 × 3 or 5 × 5) in the AFM, it was possible to detect the tip position in the optical images and calculate a transfer function that allows for compensation of the non-linearities, and aberrations in the optical images. Following that, it was possible to directly choose AFM regions of interest (ROI) from the calibrated optical images and collect data.

The correlation of the SR-SIM channels and topographical AFM data on fixed human bone osteosarcoma cell line is given in Fig. 2. Due to a significantly extended Z-scale of the AFM images (Fig. 2b), it is not always straightforward to represent the morphological information from the leading cell surface edge features demonstrated here by the cytoskeletal framework, and the significantly higher nucleus in the upper half of the AFM images. In such cases, it is beneficial to represent the AFM images by using a pixel difference Z-scale filter of some sort. In this particular case, we have treated the AFM height image in Fig. 2c with a curvature function^46^ that highlights the curvature in the image by calculating the local mean curvature operation *A* for each pixel:

1 $$A=\frac{1}{2}\left(\frac{1}{{R}_{1}}+\frac{1}{{R}_{2}}\right)$$

where *R*_1_ and *R*_2_ are the smallest and the largest radii determined at each pixel.

Combining different types of datasets, such as optical and AFM data, further has to tackle problems with the correlation and interpretation efficiency of multi-dimensional channels. On one side, it is necessary to consider that both SIM and AFM techniques carry by definition very different types of information, interpreted by the different operation principles and detection methods. This is further complicated by the different spatial and axial resolution of both techniques, which on soft biological materials can differ in the range of up to 2 (spatial) or 3 (axial) orders of magnitude. Another practical limitation is the 3D-sectioning of the samples, as well as imaging penetration depth. Whereas in the case of SR-SIM, 3D thinly sectioned images with axial resolution of down to 300 nm are now possible^47^, the Z-scaling (not resolution) in conventional AFM images is typically confined to the overall sample morphology. A certain solution to this problem is the application of force-spectroscopy based imaging (force-controlled AFM), which allows us to interpret the 3D-Force volume data of the acquired AFM dataset, i.e. each pixel has a force curve associated, and analytically subtract different quantitative information^48^. In this particular scenario, it is possible to determine the theoretical contact point in the individual force curve, and look at the sample topography or indentation information within the applied force range. This allows to look at the indentation phenomenon at a range of reference forces and enables a 3D tomographic/sectioning reconstruction of the sample within the studied indentation depth. We have given an example for that by sectioning part of the AFM image in Fig. 2b and representing the topography at different set of forces (Fig. 3).

The tip scanning AFM design further enables a simultaneous acquisition of AFM and SR-SIM images. We previously showed that for a simultaneous combination of an optical microscope equipped with a differential spinning disc and AFM, there are certain system-specific noise sources, which stem either from the AFM cantilever bimorph distortion induced by the different thermal expansion coefficients of cantilever material (*Si*/*Si*_3_*N*_4_) and reflective coatings (*Au*), or the pure mechanical noise transfer of the DSD system^8^. Our tests showed that there is no substantial difference within the noise response with the N-SIM illumination in place (Fig. 4). The results suggest that although the N-SIM illumination appears to have a small effect on the surface roughness parameters, it does not affect the acquisition or evaluation of individual structures on the cell surface. In addition, the used qp-bioAC-CI cantilevers have only a limited partial *Au* coating which considerably reduces the bending of the cantilevers during operation^8^.

## Perspectives and Future Work

Simultaneous co-localized SR-SIM/AFM data acquisition can allow visualization on how the activation or downregulation of a single MCT transporter affects the clustering at the plasma membrane. This activation can be performed with a functionalized AFM cantilever and to perform the clustering in the vicinity of the activated transporter with fluorescence sub-resolution imaging. MCT1 is encoded by the SLC16A1 gene, which belongs to the SLC16 gene family. This transmembrane protein is ubiquitously expressed in human tissues and catalyses the proton-linked transport of L-lactate, ketone bodies and pyruvate. In many types of cancers, glycolysis is preferentially utilized even under aerobic conditions, and most pyruvate is converted to lactate. In these conditions, MCT1 is reported to be overexpressed (reviewed in^49^) playing a crucial role in the import of lactate to fuel metabolism. However, the signaling events regulating the expression of MCT1 at the plasma membrane in tumour cells are still unclear. Hence, we expect that the SR-SIM/AFM system will allow to advance cell signaling investigations through the simultaneous observation of live-cell membrane transporters localization and downstream signaling processes. We aim to apply this technology to obtain simultaneously cell membrane recognition maps by QI-AFM and SR-SIM for the study of live cell dynamics, and potentially to monitor at the same time MCT1 transporter activation and downstream signaling intracellular fluorescence in the vicinity of the plasma membrane. Compared to other far-field SR fluorescence optical techniques where image acquisition speeds typically consist of multiple minutes and cause higher phototoxicity, SIM excels for long term live-cell imaging of sub-second dynamics^41^.

Recent AFM developments have demonstrated vastly increased data acquisition rates, particularly with living cells, which now allow monitoring of cytoskeletal and cell membrane dynamics on the second, and even millisecond scale^48,50,51^. Within this context, further pathways to explore on combined SR-SIM/AFM on living cells potentially harmonizing AFM and fluorescence scan rate would include HS-AFM and video-rate SIM^42^.

## Conclusions

A super-resolution SIM system has been successfully integrated with a tip-scanning QI nanomechanical mapping AFM for simultaneous SR-SIM/AFM operation. The combined SR-SIM/AFM integration was tested on 100 nm fluorescent spheres and fixed osteosarcoma cells, demonstrating a good 2D spatial correlation of both signals. The spatial resolution obtained in both cases was approaching the theoretical SIM diffraction limit. We also give an example for multidimensional representation of correlated microscopy sets, by introducing section of the 3D AFM force-volume sets. Finally, the results obtained indicate that simultaneous SR-SIM illumination during AFM cantilever operation does not distort the evaluation of the samples.

The simultaneous operation of AFM and super-resolution fluorescence microscopy technique provides a powerful observational tool on the nanoscale, albeit data acquisition is typically obstructed by a series of integration problems. We believe that the combination of SR-SIM with AFM presents one of the most promising schemes enabling simultaneous co-localized imaging, allowing the recording of nanomechanical data and cellular dynamics visualization at the same time.

## Methods

### Sample preparation

#### Fluorescent beads

Commercially available FluoSpheres beads (carboxylate-modified orange fluospheres, 0.1 *μ*m) from Thermo Fisher Scientific were used as test samples. A stock suspension containing 0.5 *μ*L fluorescent beads in 25 *μ*L of ethanol was prepared and then vortexed for 5 min. The stock solution was further diluted to 1:100 in Milli-Q water and 500 *μ*L of the beads suspension was deposited on fluorodish glass dish (bottom poly-D-Lysine coated with a glass thickness of 0.17 mm) and left to air dry at room temperature. The glass bottom dish was thoroughly cleaned on the side of the objective immersion medium with ethanol prior to measurements.

#### Cell culture and genome editing

In this work, genome-engineered cancer cells were applied to demonstrate the SR-SIM and AFM simultaneous operation. Gene-edited U2OS cells expressing monocarboxylate transporter 1 fluorescently labelled with enhanced green fluorescent protein (EGFP-MCT1) in both alleles were grown in DMEM/F12/GlutaMAX (Cat. No. 10565042, Gibco, Waltham, MA) supplemented with 10% fetal bovine serum (Cat. No. 35-010-CV, Corning) and Penicillin-Streptomycin 100 × solution (Cat. No. 15140-122, Gibco, Waltham, MA). 24 h before imaging, the cells were seeded on a *μ*-Dish 35 mm, high ibiTreat (Ibidi, Martinsried, Germany). Cells were then fixed in 4% paraformaldehyde at room temperature for 20 min and washed with Dulbecco’s phosphate-buffered saline (Cat. No. 14190-144, Gibco, Waltham, MA).

The MCT1 gene was tagged at the N-terminal using CRISPR/Cas9 gene editing tool and 5′-GTAGATAAATTCCAAAATGC-3′ as a guide RNA, both expressed utilizing pX330 plasmid^52,53^. 2 × 10^6^ U2OS cells were electroporated with 5 *μ*g of pX330 Cas9 plasmid and 15 *μ*g of donor plasmid. Cells were sorted out by an Inux sorter (BD Bioscience) for EGFP fuorescence 72 h after electroporation. Clonal populations were isolated and characterized, as previously described^54^. Sequencing revealed a one nucleotide sequence variation, which does not alter the protein or intron sequences.

### SR-SIM resolution enhancement

**Figure 5:**
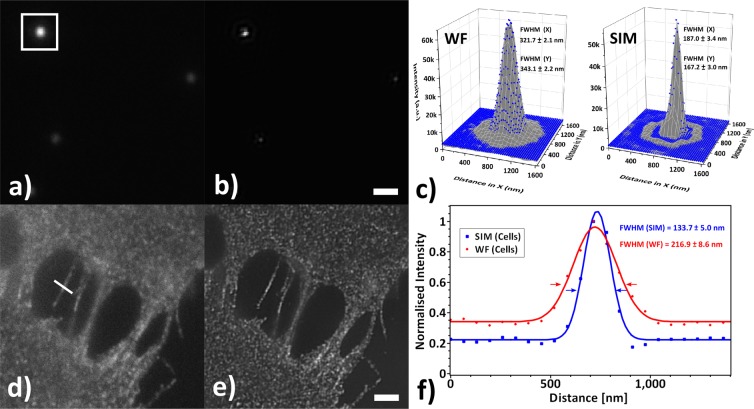
Improvement in optical resolution. (**a**) Widefield (WF) and (**b**) reconstructed images of orange fluorescent beads, 100 nm in diameter. (**d**) WF and (**e**) SR-SIM images from gene-edited U2OS cells, labelled with EGFP. The intensity of the outlined regions in (**a**,**b**) were subjected to a non-linear surface fitting with a Gaussian 2D function, allowing to compare the PSFs in (**c**). Normalized intensity profiles from the arbitrary cross-sections in (**d**,**e**) were subsequently fit with a 1D Gaussian function to allow direct correlation of the FWHM for each sample in (**f**). Scalebars in (**a**,**b**) and (**d**,**e**) correspond to 1 *μ*m and 2 *μ*m respectively.

The spatial resolution improvement of the SR-SIM system (Fig. 5) was determined using both fluorescent beads and fixed cell samples. Both widefield (WF) and SR-SIM images were acquired with a pixel size of 65 nm correspondingly at the sample plane, at starting resolution of 1024 × 1024 pixels. The positions at which arbritary cross-sections are drawn are marked in the WF panels (Fig. 5a,d). Prior to analysis, all TIFF files underwent a 16-to-8bit conversion. In addition, due to the image reconstruction algorithm used, all SR-SIM images had doubled pixel resolution (2048 × 2048), which had to be resized and downscaled to the original WF pixel resolution by a factor of 2.

The outlined areas in Fig. 5a,b were subjected to a surface fit with a Gaussian2D function (scaled Levenberg-Marquardt algorithm, tolerance of 10^−9^, resulting *R*^2^ of 0.91 for SIM and 0.99 for WF respectively) in Origin v9.6.0.172 (OriginLab Corporation, Northampton, MA), allowing for an elliptical point spread function (PSF)^55^. Following a normalization of the image intensity values in Fig. 5d,e, the cross-section profiles were fit with a Gaussian function (scaled Levenberg-Marquardt algorithm, tolerance of 10^−4^, resulting *R*^2^ of 0.99) in SciDAVis v1.22 to determine the full width at half maximum (FWHM) of the corresponding PSF. For beads we observed a reduction in the averaged FWHM of the effective focal spot from 332.4 nm to 177.1 nm, whereas for cells the FWHM was reduced from 216.9 nm to 133.7 nm, corresponding to a 1.88× and 1.62× increase in lateral resolution, approaching the theoretical maximum.

### AFM image processing

Due to the inherent sample tilt, originating from the Petri dish surfaces, all recorded AFM images were subjected to mandatory plane levelling, a procedure in which a first order polynomial surface is fitted through one or more background surface regions, and subtracted from the whole image. In addition, a median or a low-pass Gaussian filter with a sigma of not higher than 0.5–0.7 pixels in XY was only applied if some tip-induced scanning artefacts were present in the AFM images (for the applied scan dimensions and image resolutions this translates to a convolution comparable to the radius of the AFM tip). No filtering was applied in Fig. 4 which was used for quantification of any existing low-level noise from the simultaneous SIM imaging. The application of any additional specific overlay-related image enhancement filters is discussed in the text as soon as they are implemented. Image analysis was performed using the JPK Data Processing software (v6.1.120).

### Image registration

The simultaneous operation of AFM with optical microscopy enables the collection of optical sectioning fluorescence and nanomechanical mapping information from a sample. However, one complication of SIM is that, in order to avoid artefacts in the final image, requires of optimized experimental implementation, consideration of bleaching properties of the sample^56,57^ and proper selection of reconstruction parameters^58,59^, as SIM has a need of a complex post-processing step. Hence, coupling of AFM and SR-SIM can also be a powerful tool to validate the results obtained with the latter.

The successful combination of AFM with optical images is not a straightforward process. Lenses in microscopes exhibit optical aberrations that distort the image, while AFM registers “real-space” images using highly linear piezo-electric elements^60^. To circumvent this problem and correctly overlay SR-SIM data onto AFM images we use a software module (DirectOverlay, JPK BioAFM, Bruker Nano GmbH, Berlin, Germany) to calibrate the optical image. Briefly, in this calibration process the cantilever is displaced to a set of predefined coordinates in a 3 × 3 or a 5 × 5 grid pattern, and an optical image of the cantilever with an empirical estimation for the tip position is registered automatically at each position (reference points). The AFM coordinate system is treated as “real space” due to its use of closed-loop linearized piezoelectric scanners in XY (accuracy to 0.3 nm). By using the two sets of reference points, a transfer function between the AFM and the optical image is determined and this is used for the linearization of the optical images^8^, which at the end can be imported directly in the AFM software.
